# Impact of Perceived Unfairness, Uncertainty, and Life Stress on Urban Residents’ Health in China: Cross-Sectional Study

**DOI:** 10.2196/72094

**Published:** 2025-08-14

**Authors:** Jinsong Chen, Shuhan Jiang, Mingli Pang, Huan Zhou, Weifang Zhang

**Affiliations:** 1 School of Law, Hangzhou City University, Hangzhou, China; 2 School of Humanities and Management, Zhejiang Chinese Medical University, NO.548 Binwen Rd, Binjiang District, Hangzhou, 310053, China, 86 15606860005; 3 National Institute for Health Innovation, School of Population Health, University of Auckland, Auckland, New Zealand; 4 Clinical Research Center for Oral Diseases of Zhejiang Province, Key Laboratory of Oral Biomedical Research of Zhejiang Province, Cancer Center of Zhejiang University, Stomatology Hospital, School of Stomatology, School of Medicine, Zhejiang University, Hangzhou, China

**Keywords:** self-reported illness, self-reported health status, short-term illness, noncommunicable chronic diseases, unfairness stress, uncertainty stress, life stress

## Abstract

**Background:**

The prevalence of stress factors in urban settings has increased, with significant potential impact on health outcomes.

**Objective:**

This study aimed to explore the effect of perceived unfairness, uncertainty, and life stress on self-reported health status, short-term illness, and noncommunicable chronic diseases (NCDs).

**Methods:**

A cross-sectional study using multistage stratified sampling was carried out in Xi’an, Hangzhou, Guangzhou, and Guiyang in China in July 2021, and 2851 participants were included in data analysis. Self-reported health status, short-term illness, and NCDs were assessed using self-administered questionnaires. Perceived life stress, unfairness stress, and uncertainty stress were measured using a standard scale. Descriptive statistics were used to analyze participants’ demographic characteristics, and chi-square tests clarified statistical differences in self-reported health status, short-term illness, and NCDs based on these characteristics. Multiple logistic regression models were constructed to measure the effects of perceived unfairness stress, uncertainty stress, and life stress on self-reported health status, short-term illness, and NCDs.

**Results:**

Severe uncertainty stress (odds ratio [OR] 1.230, 95% CI 1.007-1.503) and severe life stress (OR 1.728, 95% CI 1.411-2.118) were associated with a higher likelihood of poor self-reported health status. Severe uncertainty stress (OR 1.565, 95% CI 1.270-1.929) and severe life stress (OR 1.404, 95% CI 1.136-1.731) increased the odds of short-term illness. In addition, severe unfairness stress (OR 1.306, 95% CI 1.053-1.620), severe uncertainty stress (OR 1.542, 95% CI 1.248-1.905), and severe life stress (OR 1.344, 95% CI 1.084-1.667) were linked to a higher prevalence of NCDs.

**Conclusions:**

To conclude, severe uncertainty and life stress were both associated with increased odds of poor health outcomes (self-reported health status, short-term illness, and NCDs), while severe unfairness only affected NCDs. The findings of this study may serve as an empirical reference for the improvement of self-reported illness among Chinese urban residents.

## Introduction

Stressors typically arise from an imbalance between an individual’s perceived needs and their ability to meet those needs. Consequently, stress occurs when individuals are unable to effectively manage or regulate these demands [1,2]. Increasing evidence suggests that stress levels among populations have reached unprecedented heights during natural disasters [3] and the COVID-19 pandemic [4], driven primarily by stressors such as a sharp economic downturn, widespread unemployment, and geopolitical instability [5].

Current research examining the relationship between stress and health has faced criticism on several fronts. According to the stress-diathesis theory, further classifications of general stress are necessary, as different types of stress exert distinct effects on health [6]; however, many studies have yet to differentiate between various forms of stress. Life stress is defined as persistent daily worries, such as poor living conditions and strained personal relationships [7,8]. These stressors have been shown to negatively affect health. A review article highlighted that life stress, especially stress arising from adverse social environments, serves as a key indicator of aging [9]. Previous studies have also demonstrated a positive association between life stress and conditions such as insomnia and depression [10,11]. Furthermore, life stress has been linked to physical health issues, including hypertension and respiratory illness [12-14].

Perceived unfairness has been identified as a significant source of stress, encompassing violations of rights or psychological infringements linked to group membership or personal experiences [15]. As a psychological construct shaped by social dynamics, perceived unfairness has profound implications for physical health. According to the perceived unfairness model, perceived injustice triggers stress responses, which can be detrimental to physical well-being [16]. Specifically, perceived unfairness contributes to anxiety and insecurity, and prolonged exposure to such psychological challenges can severely damage individuals’ social lives and well-being, ultimately impacting their overall health [17]. Substantial evidence supports the adverse effects of unfairness on various health indicators [18,19]. For instance, a study among multiethnic adults shown that perceived unfairness was related to self-reported history of depression and high blood pressure [20], Similarly, another study identified unfairness as a risk factor for metabolic syndrome and its components [21].

Uncertainty stress is a subjective experience stemming from perceptions of unpredictability and ambiguity [22]. Early studies have confirmed that uncertainty stress is a significant stressor in the context of illness and hospitalization [23], and uncertainty stress has been linked to the exacerbation of health conditions, with individuals facing health-related uncertainties reporting greater symptom severity and functional limitations [24]. Experimental research has further demonstrated that exposure to uncertainty stress leads to increases in both systolic and diastolic blood pressure [25]. Besides, chronic exposure to daily uncertainty has also been associated with a poorer quality of life and reduced well-being [26]. Given the pervasive nature of uncertainty in modern society, recent studies have shifted their focus to uncertainty stress in broader social contexts, particularly social change uncertainty and social value uncertainty [22], which reflect deeper societal and cultural transformations that shape individuals’ stress responses and health outcomes. Research has found that uncertainty stress is linked to a range of adverse health outcomes and unhealthy behaviors, including unintentional injuries [27], deliberate self-harm [28], suicidal ideation [29], problematic alcohol use [30], and poorer self-rated health [31,32]. Following the outbreak of COVID-19 pandemic, studies have demonstrated that uncertainty stress is positively associated with disease-related fear [33], depression, and anxiety symptoms [34]. However, few studies have specifically examined the relationship between uncertainty stress and different types of illnesses, highlighting a critical gap in the literature.

The novelty of this study lies in 2 key aspects. First, most previous studies on this topic have been conducted outside of China, with limited research addressing the unique characteristics of the Chinese population. Despite profound societal changes following the Reform and Opening-up, including a widening income gap and increasing disparities in resource distribution the overall perception of inequity among China’s residents appears relatively weak, with a high level of acceptance [35]. This context underscores the urgency of exploring whether perceived unfairness is still associated with adverse health outcomes and identifying the specific health outcomes involved. Second, this study uniquely explores the effects of 3 distinct stressors on health outcomes. It is critical to examine whether perceived uncertainty stress and perceived unfairness stress remain associated with health outcomes, even after controlling for life stressors. The results would be crucial for the development of future stress management strategy.

To conclude, very few studies have examined the relationship between perceived unfairness, uncertainty, and life stress in terms of urban residents’ health among the Chinese population. The aim of this study was to examine the effect of perceived unfairness, uncertainty, and life stress on urban residents’ health among the Chinese population, including self-reported illness, short-term illness, and noncommunicable chronic diseases (NCDs).

## Methods

### Participants

A cross-sectional study was conducted across 4 provincial capital cities in China—Hangzhou (East China), Xi’an (Northwest China), Guangzhou (South China), and Guiyang (West China)—in July 2021. A multistage stratified sampling approach was used. In the first stage, 4 provincial capital cities were selected to represent different geographical regions of China. In the second stage, 2 districts in the main urban area were randomly selected within each city. In the third stage, 4 communities in each selected district were chosen randomly. In the fourth stage, due to the restriction caused by the COVID-19 epidemic, convenience sampling was used within communities to recruit participants. Urban residents aged ≥18 years were eligible to enroll, and a total of 2851 participants completed the survey and were included in data analysis. Informed consent was obtained from all participants before their inclusion in the study.

### Assessment and Measurements

#### Self-Reported Health Status

Self-reported health status was measured by the item “What do you think about your health status when compared with your majority of colleagues/classmates?” The response was recoded dichotomously, with responses as excellent or good=0 and fair, poor, or bad=1 during data analysis [36].

#### Short-Term Illness

Short-term illness was assessed by the question “Have you experienced short-term illnesses such as diarrhea, colds and others at least three times in the last 6 months?” The answer was coded as “yes=1” and “no=0” [37].

#### NCDs

NCDs were assessed by the question “Are you suffering from any chronic diseases, such as digestive trouble, hypertension, other cardiovascular diseases, and others,” with the responses coded as “yes=1” and “no=0.”

#### Perceived Life Stress

Perceived life stress was measured through standard questionnaires designed by Yang and colleagues [38]: life stress from (1) unstable work and income, lack of security in life; (2) high work pressure, heavy tasks, and a fast pace; (3) tension with leaders or coworkers; (4) friction between family members; (5) difficulty in children’s education or employment; and (6) poor health of family members or oneself. These questions were measured by a 5-point Likert scale ranging from 1 (no stress) to 5 (severe stress). The average score of >3 was classified as severe life stress.

#### Perceived Unfairness Stress

Perceived unfairness stress was measured by 7 dimensions: income unfairness, education unfairness, promotion unfairness, health care unfairness, workplace benefit unfairness, pension unfairness, and other unfairness. Responses were recorded on a 5-point Likert scale from 1 to 5: never, rarely, sometimes, often, and very often. An average score more than 3 was classified as severe perceived unfairness stress.

#### Perceived Uncertainty Stress

Perceived uncertainty stress was assessed by standard questionnaires designed by Yang and colleagues [38]. The perceived uncertainty items included the following: life is unstable and cannot be controlled, uncertain about what will happen in the future, uncertain about how to achieve goals, and cannot follow social values. The score was based on a 5-point scale (very low, low, medium, high, and very high). The higher the total score, the higher the perceived uncertainty stress. An average score greater than 3 was classified as severe uncertainty stress. Previous studies have illustrated the good reliability and validity of the scale [29,30,39].

#### Sociodemographic Information

Sociodemographic characteristics of participants were measured by the following items: hukou (urban and rural; a population management policy in China), district (Xi’an, Hangzhou, Guangzhou, and Guiyang), sex (male and female), age (<25, 25‐34, 35‐44, 45‐54, >55 years), ethnicity (Han and minority), marital status (never married, married and living together, married without living together or divorced, or widowed), education (junior high school and below, senior high school, university, and postgraduate or above), occupation (government agencies, public institutions, or state-owned enterprises; private enterprise; self-employed people; freelance work; unemployed, student, or retired), average household income (1 RMB=US $0.15 at the time of the study; <50,000 RMB, 50,001‐100,000 RMB, 100,001‐150,000 RMB, 150,001‐200,000 RMB, and >200,000 RMB).

### Statistical Analysis

Descriptive statistics were used to analyze the demographic characteristics of the participants. Chi-square test was used to clarify the statistical differences of self-reported health status, short-term illness, and NCDs in terms of demographic characteristics, respectively. Multiple logistic regression models were constructed to examine the effects of perceived unfairness, uncertainty, and life stress on self-reported health status, short-term illness, and NCDs. Variables that showed statistical significance in chi-square tests were selected and then simultaneously entered into the model using the “Enter” method in SPSS (IBM Corp).

### Ethical Considerations

The study was approved by the Ethics Committee at the Zhejiang Chinese Medical University Medical Center (approval number 20210622‐3). The procedures followed were in accordance with the Declaration of Helsinki. Verbal consent was obtained from all participants before data collection, and each participant received a small gift valued at 3 yuan (1 RMB=US $0.15 at the time of the study). The study ensured that all participant data were anonymized and deidentified to protect privacy. The data were securely stored and accessible only to the research team.

## Results

As shown in Table 1, the study included 2851 participants, with a balanced representation of rural and urban hukou, a nearly equal gender distribution, and a predominance of Han ethnicity. The largest age group was participants aged <25 years (771/2851, 27.1%) and over half (1622/2851, 56.9%) were married or living together. University-educated individuals account for 46.8% (1334/2851), and 34.9% (994/2851) of them were unemployed, students, or retired. Household income levels varied, with the most common range being 150,001‐200,000 RMB (1 RMB=US $0.15 at the time of the study) per year. Notable disparities in health outcomes were observed across different sociodemographic participants. Rural hukou individuals reported poorer self-reported health status (485/1382, 35.1%) compared to urban hukou individuals (420/1469, 28.6%), while urban hukou individuals exhibited a slightly higher prevalence of NCDs. Significant regional differences were also noted, with Xi’an reporting the highest prevalence of both poor self-reported health status and NCDs. Age was a strong determinant, with older participants (>55 y) showing higher prevalence of poor self-reported health status and NCDs. Other factors, such as ethnicity, education level, and employment status, were similarly associated with variations in health outcomes.

**Table 1.** Sociodemographic characteristics of urban residents in Xi’an, Hangzhou, Guangzhou, Guiyang, China, in July 2021.

Variables	Values, n (%)	Experiences and prevalence		Chi-square (*df*), *P* value	
		Poor self-reported health status, n (%)	NCDs^a^, n (%)	Self-reported health status	NCDs
Hukou				13.899 (1), *P*<.001	1.414 (1), *P*=.234
Urban	1469 (51.5)	420 (28.6)	479 (32.6)		
Rural	1382 (48.5)	485 (35.1)	422 (30.5)		
District				29.876 (3), *P*<.001	34.093 (3), *P*<.001
Xi’an	770 (27)	267 (34.7)	292 (37.9)		
Hangzhou	699 (24.5)	223 (31.9)	242 (34.6)		
Guangzhou	835 (29.3)	293 (35.1)	215 (25.7)		
Guiyang	547 (19.2)	122 (22.3)	152 (27.8)		
Sex				1.849 (1), *P*=.174	2.123 (1), *P*=.145
Male	1411 (49.5)	431 (30.5)	464 (32.9)		
Female	1440 (50.5)	474 (32.9)	437 (30.3)		
Age (years)				42.824 (4), *P*<.001	292.324 (4), *P*<.001
<25	771 (27.1)	236 (30.6)	142 (18.4)		
25‐34	443 (15.5)	112 (25.3)	83 (18.7)		
35‐44	581 (20.4)	162 (27.9)	166 (28.6)		
45‐54	485 (17)	153 (31.5)	177 (36.5)		
>55	571 (20)	242 (42.4)	333 (58.3)		
Ethnicity				17.564 (1), *P*<.001	4.299 (1), *P*=.038
Han	2436 (85.4)	810 (33.3)	788 (32.3)		
Minority	415 (14.6)	95 (22.9)	113 (27.2)		
Marital status				34.018 (3), *P*<.001	171.839 (3), *P*<.001
Never married	1000 (35.1)	293 (29.3)	189 (18.9)		
Married, and living together	1622 (56.9)	506 (31.2)	588 (36.3)		
Married without living together or divorced	126 (4.4)	48 (38.1)	50 (39.7)		
Widowed	103 (3.6)	58 (56.3)	74 (71.8)		
Education				45.357 (3), *P*<.001	170.152 (3), *P*<.001
Junior high school and below	675 (23.7)	280 (41.5)	347 (51.4)		
Senior high school	683 (24)	172 (25.2)	204 (29.9)		
University	1334 (46.8)	410 (30.7)	308 (23.1)		
Postgraduate or above	159 (5.5)	43 (27)	42 (26.4)		
Occupation				50.543 (4), *P*<.001	39.179 (4), *P*<.001
Government agencies, public institutions, or state-owned enterprises	467 (16.4)	110 (23.6)	175 (37.5)		
Private enterprise	469 (16.5)	135 (28.8)	99 (21.1)		
Self-employed people	404 (14.1)	118 (29.2)	130 (32.2)		
Freelance work	517 (18.1)	146 (28.2)	148 (28.6)		
Unemployed, student, or retired	994 (34.9)	396 (39.8)	349 (35.1)		
Average household income (RMB)^b^				52.368 (4), *P*<.001	25.876 (4), *P*<.001
<50,000	793 (27.8)	324 (40.9)	294 (37.1)		
50,001‐100,000	865 (30.4)	277 (32.0)	288 (33.3)		
100,001‐150,000	608 (21.3)	155 (25.5)	168 (27.6)		
150,001‐200,000	280 (9.8)	68 (24.3)	75 (26.8)		
>200,000	305 (10.7) 81	224 (26.6)	76 (24.9)		

a NCDs: noncommunicable chronic diseases.

b 1 RMB=US $0.15 at the time of the study.

As shown in Table 2, multiple logistic regression models were constructed to estimate the odds ratios (ORs) for self-reported health status, adjusting for unfairness stress, uncertainty stress, and life stress, as well as a full model including all variables. Key findings revealed that rural hukou individuals had higher odds of poor self-reported health compared to urban hukou individuals. Significant regional differences were also observed, with Hangzhou and Guangzhou showing higher odds of poor health compared to Xi’an, while the difference between Guiyang and Xi’an was not statistically significant. Age was also a significant factor, with individuals aged >55 years having the highest odds of poor health. Sociodemographic factors, such as ethnicity, marital status, education level, occupation, and income, also played critical roles.

**Table 2.** Multiple logistic regression models of self-reported health status among urban residents in Xi’an, Hangzhou, Guangzhou, or Guiyang, China, in July 2021.

Group	Model 1: Unfairness model	Model 2: Uncertainty stress model	Model 3: Life stress model	Model 4: Full model, OR
Hukou, OR^a^ (95% CI)				
Urban	1.00 (reference)	1.00 (reference)	1.00 (reference)	1.00 (reference)
Rural	1.243 (1.036-1.491)^b^	1.229 (1.024-1.475)^b^	1.204 (1.002-1.446)^b^	1.205(1.003-1.449)^b^
District, OR (95% CI)				
Xi’an	1.00 (reference)	1.00 (reference)	1.00 (reference)	1.00 (reference)
Hangzhou	1.291 (1.008-1.655)^b^	1.296 (1.011-1.661)^b^	1.428 (1.110-1.837)^c^	1.442 (1.120-1.856)^c^
Guangzhou	1.693 (1.335-2.146)^c^	1.705 (1.344-2.162)^c^	1.738 (1.369-2.208)^c^	1.733 (1.364-2.202)^c^
Guiyang	0.770 (0.566-1.049)	0.755 (0.555-1.026)	0.760 (0.558-1.033)	0.798 (0.584-1.091)
Age (years), OR (95% CI)				
<25	1.00 (reference)	1.00 (reference)	1.00 (reference)	1.00 (reference)
25‐34	0.921 (0.648-1.310)	0.957 (0.673-1.363)	0.932 (0.653-1.330)	0.934 (0.654-1.333)
35‐44	1.038 (0.713-1.511)	1.075 (0.737-1.568)	1.081 (0.740-1.578)	1.070 (0.732-1.564)
45‐54	1.214 (0.821-1.793)	1.258 (0.850-1.861)	1.231 (0.830-1.825)	1.242 (0.837-1.843)
>55	1.625 (1.107,2.385)^b^	1.758 (1.195,2.587)^c^	1.860 (1.261,2.742)^c^	1.859 (1.259,2,746)^c^
Ethnicity, OR (95% CI)				
Han	1.00 (reference)	1.00 (reference)	1.00 (reference)	1.00 (reference)
Minority	0.819 (0.609-1.102）	0.803 (0.596-1.082)	0.799 (0.593-1.078)	0.786 (0.582-1.060)
Marital status, OR (95% CI)				
Never married	1.00 (reference)	1.00 (reference)	1.00 (reference)	1.00 (reference)
Married, and living together	1.457 (1.060-2.003)^b^	1.425 (1.037-1.960)^b^	1.437 (1.043-1.980)^b^	1.422 (1.032-1.960)^b^
Married without living together or divorced	2.293 (1.419-3.705)^c^	2.219 (1.373-3.584)^c^	2.184 (1.349-3.534)^c^	2.160 (1.334-3.496)^c^
Widowed	3.021 (1.786-5.112)^c^	2.917 (1.718-4.953)^c^	2.933 (1.723-4.993)^c^	2.909 (1.706-4.960)^c^
Education, OR (95% CI)				
Junior high school and below	1.00 (reference)	1.00 (reference)	1.00 (reference)	1.00 (reference)
Senior high school	0.727 (0.561-0.942)^b^	0.743 (0.573-0.964)^b^	0.720 (0.555-0.934)^b^	0.730 (0.562-0.947)^b^
University	1.114 (0.853-1.456）	1.129 (0.864-1.476)	1.097 (0.838-1.435)	1.099 (0.839-1.439)
Postgraduate or above	1.047 (0.664-1.651)	1.074 (0.681-1.695)	1.045 (0.661-1.654)	1.054 (0.666-1.669)
Occupation, OR (95% CI)				
Government agencies, public institutions, or state-owned enterprises	1.00 (reference)	1.00 (reference)	1.00 (reference)	1.00 (reference)
Private enterprise	1.370 (0.997-1.881)	1.344 (0.979-1.845)	1.371 (0.997-2.106)	1.383 (1.005-1.904)^b^
Self-employed people	1.467 (1.052-2.046)^b^	1.433 (1,027-1.999)^b^	1.426 (1.020-1.994)^b^	1.429 (1.022-1.998)^b^
Freelance work	1.247 (0.911-1.707)	1.229 (0.897-1.684)	1.248 (0.909-1.713)	1.241 (0.904-1.704)
Unemployed, student, or retired	2.112 (1.559-2.861)^c^	2.005 (1.479-2.718)^c^	2.083 (1.533-2.829)^c^	2.064 (1.518-2.806)^c^
Average household income (RMB)^d^, OR (95% CI)				
<50,000	1.00 (reference)	1.00 (reference)	1.00 (reference)	1.00 (reference)
50,001‐100,000	0.798 (0.640-0.996)^b^	0.805 (0.645-1.004)	0.808 (0.647-1.010)	0.812 (0.650-1.015)
100,001‐150,000	0.619 (0.475-0.806)^c^	0.624 (0.479-0.812)^c^	0.618 (0.474-0.807)^c^	0.626 (0.480-0.817)^c^
150,001‐200,000	0.548 (0.387-0.775)^c^	0.549 (0.388-0.776)^c^	0.553 (0.390-0.784)^c^	0.561 (0.396-0.795)^c^
>200,000	0.552 (0.397-0.769)^c^	0.556 (0.399-0.775)^c^	0.561 (0.402-0.782)^c^	0.565 (0.405-0.790)^c^
Unfairness stress, OR (95% CI)				
Low unfairness stress	1.00 (reference)	—^e^	—	1.00 (reference)
Severe unfairness stress	1.490 (1.248-1.779)^c^	—	—	1.069 (0.871-1.313)
Uncertainty stress, OR (95% CI)				
Low uncertainty stress	—	1.00 (reference)	—	1.00 (reference)
Severe uncertainty stress	—	1.620 (1.368-1.919)^c^	—	1.230 (1.007-1.503)^b^
Life stress, OR (95% CI)				
Low life stress	—	—	1.00 (reference)	1.00 (reference)
Severe life stress	—	—	1.959 (1.648-2.330)^c^	1.728 (1.411-2.118)^c^
Fixed parameters, β	0.180^c^	0.163^c^	0.149^c^	0.138^c^

a OR: odds ratio.

b *P*<.05.

c *P*<.01.

d 1 RMB=US $0.15 at the time of the study.

e Not applicable.

Stress factors were important determinants, and individuals who were at severe uncertainty stress and life stress had significantly higher the odds of poor health. Both were statistically significant.

Multiple logistic regression models (short-term illness) were constructed to estimate the ORs, adjusting for unfairness stress, uncertainty stress, life stress, and a full model incorporating all factors. As shown in Table 3, key findings highlighted that rural hukou individuals had slightly higher odds (OR 1.206, 95% CI 0.996-1.460) of short-term illness compared to urban hukou individuals, and regional disparities were evident. Compared to Xi’an, Hangzhou showed lower odds of short-term illness, while Guangzhou and Guiyang exhibited little to no difference. Sociodemographic factors such as age, ethnicity, marital status, education, and income also played roles.

**Table 3.** Multiple logistic regression models of short-term illness among urban residents in Xi’an, Hangzhou, Guangzhou, or Guiyang, China, in July 2021.

Group	Model 1: Unfairness model	Model 2: Uncertainty stress model	Model 3: Life stress model	Model 4: Full model
Hukou, OR^a^ (95% CI)				
Urban	1.00 (reference)	1.00 (reference)	1.00 (reference)	1.00 (reference)
Rural	1.240 (1.026-1.500)^b^	1.224 (1.011-1.481)^b^	1.208 (0.998-1.462)	1.206 (0.996-1.460)
District, OR (95% CI)				
Xi’an	1.00 (reference)	1.00 (reference)	1.00 (reference)	1.00 (reference)
Hangzhou	0.613 (0.469-0.799)^c^	0.627 (0.480-0.819)^c^	0.660 (0.505-0.863)^c^	0.669 (0.511-0.876)^b^
Guangzhou	0.992 (0.777-1.267)	0.999 (0.781-1.277)	1.011 (0.791-1.293)	1.008 (0.788-1.289)
Guiyang	0.993 (0.732-1.384)	1.016 (0.749-1.379)	0.973 (0.719-1.318)	1.046 (0.768-1.427)
Age (years), OR (95% CI)				
<25	1.00 (reference)	1.00 (reference)	1.00 (reference)	1.00 (reference)
25‐34	0.869 (0.612-1.235)	0.896 (0.629-1.276)	0.877 (0.616-1.248)	0.884 (0.619-1.260)
35‐44	0.801 (0.548-1.170)	0.820 (0.560-1.203)	0.829 (0.567-1.214)	0.823 (0.561-1.208)
45‐54	0.681 (0.454-1.019)	0.706 (0.470-1.060)	0.690 (0.459-1.036)	0.702 (0.467-1.056)
>55	0.999 (0.677-1.474)	1.097 (0.741-1.626)	1.123 (0.758-1.662)	1.144 (0.770-1.699)
Ethnicity, OR (95% CI)				
Han	1.00 (reference)	1.00 (reference)	1.00 (reference)	1.00 (reference)
Minority	0.869 (0.646-1.167)	0.829 (0.614-1.118)	0.849 (0.630-1.143)	0.817 (0.604-1.103)
Marital status, OR (95% CI)				
Never married	1.00 (reference)	1.00 (reference)	1.00 (reference)	1.00 (reference)
Married, and living together	0.981 (0.714-1.347)	0.951 (0.691-1.308)	0.963 (0.700-1.324)	0.942 (0.684-1.297)
Married without living together or divorced	1.435 (0.882-2.334)	1.378 (0.846-2.245)	1.371 (0.842-2.233)	1.345 (0.825-2.193)
Widowed	1.905 (1.138-3.189)^b^	1.823 (1.084-3.066)^b^	1.835 (1.092-3.082)^b^	1.795 (1.065-3.024)^b^
Education, OR (95% CI)				
Junior high school and below	1.00 (reference)	1.00 (reference)	1.00 (reference)	1.00 (reference)
Senior high school	0.612 (0.466-0.804)^c^	0.626 (0.476-0.824)^c^	0.605 (0.460-0.794)^c^	0.619 (0.470-0.814)^c^
University	0.752 (0.570-0.991)^b^	0.761 (0.577-1.004)	0.742 (0.562-0.979)^b^	0.747 (0.566-0.987)^b^
Postgraduate or above	0.672 (0.418-1.080)	0.687 (0.426-1.108)	0.676 (0.420-1.088)	0.683 (0.423-1.102)
Occupation, OR (95% CI)				
Government agencies, public institutions, or state-owned enterprises	1.00 (reference)	1.00 (reference)	1.00 (reference)	1.00 (reference)
Private enterprise	1.006 (0.725-1.395)	0.986 (0.709-1.369)	1.002 (0.722-1.390)	1.001 (0.720-1.391)
Self-employed people	1.191 (0.849-1.670)	1.159 (0.825-1.628)	1.160 (0.826-1.630)	1.153 (0.820-1.622)
Freelance work	0.841 (0.607-1.165)	0.818 (0.589-1.136)	0.838 (0.604-1.162)	0.822 (0.591-1.142)
Unemployed, student, or retired	1.105 (0.812-1.504)	1.035 (0.759-1.411)	1.083 (0.794-1.476)	1.047 (0.767-1.430)
Average household income (RMB)^d^, OR (95% CI)				
<50,000	1.00 (reference)	1.00 (reference)	1.00 (reference)	1.00 (reference)
50,001‐100,000	1.015 (0.804-1.282)	1.028 (0.813-1.299)	1.027 (0.812-1.298)	1.036 (0.819-1.310)
100,001‐150,000	1.018 (0.774-1.337)	1.035 (0.786-1.361)	1.016 (0.772-1.336)	1.037 (0.787-1.366)
150,001‐200,000	1.055 (0.740-1.505)	1.069 (0.748-1.526)	1.055 (0.739-1.507)	1.078 (0.754-1.541)
>200,000	0.912 (0.642-1.295)	0.929 (0.653-1.323)	0.921 (0.648-1.310)	0.939 (0.659-1.337)
Unfairness stress, OR (95% CI)				
Low unfairness stress	1.00 (reference)	—^e^	—	1.00 (reference)
Severe unfairness stress	1.436 (1.194-1.727)^c^	—	—	1.012 (0.817-1.253)
Uncertainty stress, OR (95% CI)				
Low uncertainty stress	—	1.00 (reference)	—	1.00 (reference)
Severe uncertainty stress	—	1.834 (1.535-2.191)^c^	—	1.565 (1.270-1.929)^c^
Life stress, OR (95% CI)				
Low life stress	—	—	—	1.00 (reference)
Severe life stress	—	—	1.738 (1.453-2.078)^c^	1.404 (1.136-1.731)^c^
Fixed parameters, β	0.429^c^	0.358^c^	0.377^c^	0.327^c^

a OR: odds ratio.

b *P*<.05.

c *P*<.01.

d 1 RMB=US $0.15 at the time of the study.

e Not applicable.

Individuals experiencing severe uncertainty stress had a 56.5% higher odds of reporting short-term illness compared to those with low uncertainty stress. Individuals experiencing severe life stress had 40.4% higher odds of reporting short-term illness compared to those with low life stress. Both were statistically significant.

Multiple logistic regression models (NCD) were constructed to estimate ORs, adjusting for unfairness stress, uncertainty stress, life stress, and a full model incorporating all factors.

Illustrated in Table 4, key findings showed that rural hukou individuals had a slightly lower odds of NCDs compared with urban hukou individuals. Significant regional differences were observed, with Hangzhou reporting higher odds (OR 1.290, 95% CI 0.994‐1.674) of NCDs compared to Xi’an, while Guiyang showed lower odds. Age was also a significant factor, with older participants, especially those over 55 years old, having the highest OR of NCDs. Other sociodemographic variables like ethnicity, marital status, education level, occupation, and income also influenced outcomes.

**Table 4.** Multiple logistic regression models of noncommunicable chronic diseases among urban residents in Xi’an, Hangzhou, Guangzhou, or Guiyang, China, in July 2021.

Group	Model 1: Unfairness model	Model 2: Uncertainty model	Model 3: Life stress model	Model 4: Full model
Hukou, OR^a^ (95% CI)				
Urban	1.00 (reference)	1.00 (reference)	1.00 (reference)	1.00 (reference)
Rural	0.825 (0.684-0.995)	0.797 (0.659-0.963)	0.795 (0.659-0.960)	0.797 (0.659-0.963)
District, OR (95% CI)				
Xi’an	1.00 (reference)	1.00 (reference)	1.00 (reference)	1.00 (reference)
Hangzhou	1.092 (0.858-1.391)	1.095 (0.859-1.394)	1.155 (0.904-1.475)	1.290 (0.994-1.674)
Guangzhou	0.983 (0.769-1.256)	0.996 (0.779-1.274)	1.012 (0.792-1.294)	1.054 (0.820-1.356)
Guiyang	0.735 (0.542-0.996)	0.708 (0.523-0.958)	0.670 (0.496-0.904)	0.803 (0.587-1.098)
Age (years), OR (95% CI)				
<25	1.00 (reference)	1.00 (reference)	1.00 (reference)	1.00 (reference)
25‐34	0.999 (0.678-1.471)	1.045 (0.707-1.543)	1.030 (0.698-1.522)	1.015 (0.687-1.501)
35‐44	1.440 (0.968-2.141)	1.494 (1.001-2.229)^b^	1.533 (1.029-2.284)^b^	1.476 (0.988-2.204)
45‐54	1.821 (1.211-2.738)^b^	1.899 (1.258-2.866)^b^	1.858 (1.232-2.800)^b^	1.892 (1.253-2.857)^b^
>55	3.479 (2.330-5.194)^c^	3.879 (2.586-5.821)^c^	3.972 (2.659-5.956)^c^	3.965 (2.637-5.963)^c^
Ethnicity, OR (95% CI)				
Han	1.00 (reference)	1.00 (reference)	1.00 (reference)	1.00 (reference)
Minority	0.956 (0.712-1.284)	0.932 (0.693-1.254)	0.956 (0.712-1.285)	0.918 (0.682-1.234)
Marital status, OR (95% CI)				
Never married	1.00 (reference)	1.00 (reference)	1.00 (reference)	1.00 (reference)
Married, and living together	1.363 (0.979-1.897)	1.339 (0.960-1.866)	1.356 (0.973-1.889)	1.331 (0.953-1.857)
Married without living together or divorced	2.161 (1.328-3.516)^b^	2.089 (1.283-3.401)^b^	2.108 (1.298-3.425)^b^	2.049 (1.258-3.339)^b^
Widowed	3.413 (1.949-5.977)^c^	3.322 (1.889-5.844)^c^	3.348 (1.906-5.880)^c^	3.336 (1.894-5.877)^c^
Education, OR (95% CI)				
Junior high school and below	1.00 (reference)	1.00 (reference)	1.00 (reference)	1.00 (reference)
Senior high school	0.615 (0.479-0.791)^c^	0.636 (0.494-0.819)^c^	0.609 (0.473-0.783)^c^	0.628 (0.487-0.809)^c^
University	0.549 (0.423-0.713)^c^	0.562(0.433-0.731)^c^	0.545 (0.420-0.708)^c^	0.550 (0.423-0.716)^c^
Postgraduate or above	0.508 (0.325-0.795)^b^	0.534 (0.341-0.836)	0.509 (0.325-0.7981)^c^	0.525 (0.334-0.825)
Occupation, OR (95% CI)				
Government agencies, public institutions, or state-owned private enterprises	1.00 (reference)	1.00 (reference)	1.00 (reference)	1.00 (reference)
Private enterprise	0.596 (0.435-0.817)^c^	0.574 (0.419-0.788)^c^	0.574 (0.419-0.787)^c^	0.589 (0.429-0.810)^c^
Self-employed people	0.753 (0.550-1.030)	0.725 (0.529-0.994)^b^	0.726 (0.530-0.995)^b^	0.738 (0.537-1.013)
Freelance work	0.597 (0.442-0.807)^c^	0.594 (0.438-0.805)^c^	0.603 (0.445-0.816)	0.595 (0.439-0.807)^c^
Unemployed, student, or retired	1.047 (0.779-1.408)	0.966 (0.716-1.302)	1.021 (0.758-1.375)	0.989 (0.733-1.336)
Average household income (RMB)^d^, OR (95% CI)				
<50,000	1.00 (reference)	1.00 (reference)	1.00 (reference)	1.00 (reference)
50,001‐100,000	0.586 (0.426-0.806)^b^	0.567 (0.412-0.780)^c^	0.564 (0.410-0.776)^c^	0.582 (0.422-0.802)^b^
100,001‐150,000	0.762 (0.556-1.044)	0.733 (0.534-1.006)	0.734 (0.535-1.006)	0.740 (0.539-1.017)
150,001‐200,000	0.617 (0.456-0.835)^b^	0.605 (0.446-0.820)^b^	0.614 (0.453-0.832)^b^	0.606 (0.446-0.823)^b^
>200,000	1.036 (0.769-1.396)	0.957 (0.709-1.292)	1.006 (0.745-1.357)	0.985 (0.728-1.331)
Unfairness stress, OR (95% CI)				
Low unfairness stress	1.00 (reference)	—^e^	—	1.00 (reference)
Severe unfairness stress	1.813 (1.505-2.183)^c^	—	—	1.306 (1.053-1.620)^b^
Uncertainty stress, OR (95% CI)				
Low uncertainty stress	—	1.00 (reference)	—	1.00 (reference)
Severe uncertainty stress	—	1.971 (1.648-2.357)^c^	—	1.542 (1.248-1.905)^c^
Life stress, OR (95% CI)				
Low life stress	—	—	1.00 (reference)	1.00 (reference)
Severe life stress	—	—	1.848 (1.540-2.216)^c^	1.344 (1.084-1.667)^c^
Fixed parameters, β	0.376^c^	0.334^c^	0.353^c^	0.293^c^

a OR: odds ratio.

b *P*<.05.

c *P*<.01.

d 1 RMB=US $0.15 at the time of the study.

e Not applicable.

Individuals experiencing severe unfairness stress had 30.6% higher odds of developing NCDs compared to those with low uncertainty stress. Those with severe uncertainty stress had 54.2% higher odds and those with severe life stress had 34.4% higher odds. All findings were statistically significant.

## Discussion

### Principal Findings

The study provided valuable insights into self-reported illness among urban residents in China, particularly concerning perceptions of unfairness, uncertainty, and life stress. Furthermore, this research pioneered an exploration of how perceived unfairness, uncertainty, and life stress relate to self-reported illness, encompassing health conditions such as short-term illness and NCDs.

### The Effect of Perceived Unfairness, Uncertainty, and Life Stress on Health Status

Individuals experiencing severe uncertainty (OR 1.230, 95% CI 1.007-1.503) and life stress (OR 1.728, 95% CI 1.411-2.118) were more likely to report health problems, due to the physiological and psychological impacts of prolonged stress [40]. Uncertainty intensified anxiety levels, impeding effective coping strategies and exacerbating stress responses [41]. Chronic life stress contributed to dysregulation of the hypothalamic-pituitary-adrenal axis [42], leading to prolonged secretion of stress hormones like cortisol, increase vulnerability to illnesses [43], contributing to poor self-reported health. Moreover, prolonged stress could disrupt sleep patterns [44], appetite regulation [45], and cardiovascular health [46], further compromising overall well-being.

### The Effect of Perceived Unfairness, Uncertainty, and Life Stress on Short-Term Illness

Individuals experiencing severe uncertainty stress (OR 1.565, 95% CI 1.270-1.929) and life stress (OR 1.404, 95% CI 1.136-1.731) were more likely to report higher odds of short-term illness. Uncertainty stress significantly elevated anxiety, disrupted coping mechanisms, and impaired immune function. The heightened stress led to prolonged activation of the hypothalamic-pituitary-adrenal axis [42], resulting in elevated cortisol levels, then altering the gut microbiota composition, weakening the gut barrier and increasing the risk of infections. Furthermore, chronic life stress could exacerbate gut inflammation [47], dysbiosis [48], and disrupt gut motility and transit time, all of these were associated with short-term illness.

### The Effect of Perceived Unfairness, Uncertainty, and Life Stress on NCDs

Individuals experiencing severe unfairness (OR 1.306, 95% CI 1.053-1.620), uncertainty (OR 1.542, 95% CI 1.248-1.905), and life stress (OR 1.344, 95% CI 1.084-1.667) exhibited significantly higher odds of NCDs. These stressors contributed to the development of NCDs through a variety of physiological and psychological mechanisms [49]. Unfairness stress could trigger chronic systemic inflammation by activating the release of pro-inflammatory cytokines, which were implicated in the pathogenesis of conditions like cardiovascular disease and diabetes [50]. Moreover, unfairness stress could disrupt metabolic homeostasis, leading to dysregulated glucose metabolism and insulin resistance, further predisposing individuals to diabetes and metabolic syndrome [51].

Uncertainty stress would lead to prolonged stress response: it not only impaired immune function but also promoted oxidative stress and inflammation, contributing to the development of NCDs. Dysregulation of the immune system under uncertainty stress could also impair immune surveillance against cancer cells and increase susceptibility to infections [52], potentially influencing the onset and progression of various NCDs. Additionally, chronic stress disrupted sleep patterns [44] and might lead to unhealthy coping behaviors [53], such as poor diet choices, physical inactivity, and substance abuse, all of which are risk factors for NCDs.

Although some public health professionals and members of the public recognize that perceived stress is associated with adverse health outcomes [54,55], few studies have specifically examined the impact of different stressors on various types of illness. A key finding of this study is that even after controlling for life stress and perceived uncertainty stress, perceived unfairness stress remains significantly associated with a higher prevalence of NCDs. We believe this is closely linked to China’s unique sociocultural context. Hofstede’s cultural dimension theory identifies China as one of the nations with the highest power distance indices globally [56]. In such a culture, the unequal distribution of power and resources is often regarded as natural and legitimate, leading individuals to internalize power disparities and normalize unfair social relations [57]. It is reported that Chinese residents tend to have a limited perception of unfairness and a high level of acceptance [35]. However, despite this apparent adaptation to unfairness, such dignitary harm [58] threatens the fundamental basis of social respect [59]. Living in a society with low levels of fairness negatively affects psychological well-being by diminishing feelings of social justice, reducing social trust, and weakening reciprocity [60]. As a result, individuals face greater constraints in accessing coping resources, which, according to Conservation of Resources theory, may lead to chronic and long-term harm to physical health [61].

### Implications

First, implement programs that provide psychosocial support tailored to address perceived unfairness, uncertainty, and life stress among urban residents, include counseling services, stress management workshops, and community support groups to enhance mental resilience. Second, encourage employers to establish stress management initiatives in workplaces, focusing on mitigating factors such as perceived unfairness and uncertainty, like promoting work-life balance, improving communication channels, and offering employee assistance programs to support mental well-being. Third, community mental health programs should address uncertainty stress through targeted interventions, such as digital and online resources, public awareness campaigns, tailored interventions targeting high-risk groups, as well as crisis helplines.

### Limitations

First, this study was a cross-sectional study, and could not clearly indicate the causal relationship between the variables. However, this study was large-scale, and results could provide some implications regarding the important associations between different types of stress and urban residents’ health. Second, in this study, an important outcome variable was “Are you suffering from chronic diseases?” No in-depth research was conducted to investigate the type and severity of NCDs, but the data collected still offered a general overview of the prevalence of chronic conditions among the surveyed population, offering valuable insights for policy makers and contributing to a better understanding of the health outcomes of stress. Third, as this study focused on specific urban residents in China, the results might not fully capture the experiences or health outcomes of other regions, which would limit the broader applicability of the conclusions, but it is still useful for other countries at a similar stage of development as China. Finally, the reliance on self-reported health outcomes, which were not validated using clinical measures, may introduce reporting bias due to participants’ subjective interpretations or recall errors; however, the use of self-reported data is a widely used method in public health research for capturing individual health awareness and perceived well-being.

### Conclusions

In summary, the cross-sectional study clarified the effect of perceived unfairness, uncertainty, and life stress on self-reported illness among Chinese urban residents. The study found significant associations between several stress factors and self-reported health status. Severe uncertainty and life stress were both associated with increased odds of poor health outcomes, while severe unfairness was only associated with NCDs. Future longitudinal research is needed to better understand the temporal dynamics and causal pathways underlying these associations and to further inform effective interventions that decrease stress and promote well-being in urban populations. The findings of this study emphasize the importance of policies that strive for a fairer society, with a great focus on well-being, and may serve as an empirical reference for improving population health among Chinese urban residents.

## Abbreviations

NCD: noncommunicable chronic diseases
OR: odds ratio
